# Effects of levosimendan on mortality in patients with septic shock: systematic review with meta-analysis and trial sequential analysis

**DOI:** 10.18632/oncotarget.20123

**Published:** 2017-08-10

**Authors:** Benji Wang, Rujie Chen, Xianyang Guo, Wenwu Zhang, Jianjian Hu, Yuqiang Gong, Bihuan Cheng

**Affiliations:** ^1^ Department of Critical Care Medicine, The Second Affiliated Hospital and Yuying Children’s Hospital of Wenzhou Medical University, Wenzhou 325000, Zhejiang, China

**Keywords:** levosimendan, mortality, septic shock, meta-analysis, trial sequential analysis

## Abstract

**Object:**

Several studies have investigated a survival benefit for levosimendan treatment in patients with septic shock. However, data are conflicting. We conducted a meta-analysis to evaluate the effect of levosimendan treatment on mortality in patients with septic shock.

**Materials and Methods:**

We searched PubMed, EMBASE and Cochrane Library Databases up to March 27, 2017, without language restrictions. We searched for terms related to septic shock, levosimendan, randomized clinical trial. Randomized controlled trials reported the effect of levosimendan on mortality were included. Moreover, we constructed the trial sequential analysis (TSA) to determine the reliability of the outcomes. Furthermore, secondary outcomes were cardiac index(CI), mean arterial pressure (MAP), blood lactate, norepinephrine dose and length of ICU stay.

**Results:**

Ten studies with a total of 816 patients were included in this meta-analysis. There was no significant difference in the mortality between the levosimendan group and the standard inotropic therapy group [RR = 0.96, 95% CI (0.81–1.12), *I*^2^ = 0]. However, methods adapted from formal interim monitoring boundaries applied to TSA indicated that the cumulative evidence was unreliable and inconclusive. Blood lactate was significantly reduced in the levosimendan group while there was no difference in MAP, CI, norepinephrine dose and length of ICU stay.

**Conclusions:**

Findings from this meta-analysis demonstrated that levosimendan treatment may not reduce mortality in patients with septic shock. The result remains inclusive and further randomized controlled trials were needed to confirm these conclusions.

## INTRODUCTION

Sepsis is defined as life-threatening organ dysfunction caused by a dysregulated host response to infection. [1] Septic shock is the most severe form of the condition and results in circulatory and metabolic abnormalities [2]. The mortality and morbidity rate of patients with septic shock has remained high [3, 4], despite decades of medical advances [5]. Persisting hypotension despite adequate fluid resuscitation is due to a combination of profound vasodilatation, vascular hyporeactivity to catecholamine, and myocardial depression [6]. Cardiovascular dysfunction owing to severe infection is thought to play an important role in sepsis related mortality [7].

Dobutamine is widely acknowledged that can be used in the treatment of septic cardiomyopathy, as international sepsis guidelines recommended [5]. A body of epidemiologic studies have demonstrated that the use of dobutamine increases contractility and cardiac output, but it does not improve microcirculation or peripheral perfusion [8], and even increased the mortality rate [9].

Levosimendan is a calcium-sensitizing drug with inotropic, which has been used for treatment of decompensated heart failure [10]. It increases myocardial contractility with vasodilatory properties [11], meanwhile diastolic relaxation is not impaired [12]. Several studies have reported that levosimendan exerts anti-ischemic, anti-inflammatory, and anti-apoptotic properties, thereby affecting important pathways in the pathophysiology of septic shock [13–16]. Furthermore, it also has shown improvements in hemodynamic variables, microcirculatory flow, and renal and hepatic function, as compared with dobutamine [17–19]. A recent meta-analysis indicated that levosimendan was associated with significantly reduced risk of death as compared to the conventional inotropes [20]. A recent review article also illustrated that Levosimendan might be potentially beneficial in reducing mortality risk [21]. However, one recently large trial demonstrated controversial result. The result did not show any survival benefit for levosimendan treatment in patients with septic shock [22].

So we performed a comprehensive systematic review and meta-analysis of randomized clinical trials to assess the effects of levosimendan on mortality in patients with septic shock.

## RESULTS

### Literature search

**Figure 1:**
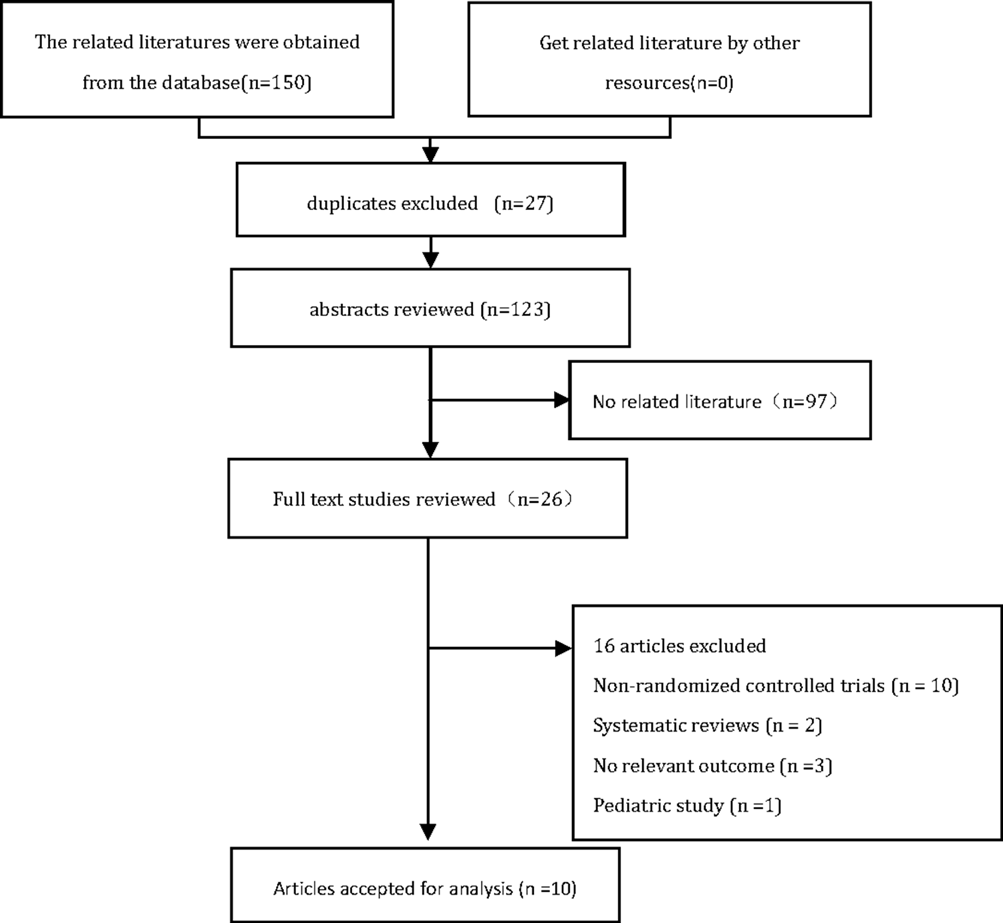
PRISMA flow diagram for trial selection

According to the search strategy, a total of 150 related studies were retrieved. Removing duplicate studies and evaluating the quality of the literatures, ten RCTs (816 participants) were eventually included in the study [17–19, 22–28]. The flow diagram summarizing the process of study selection is shown in Figure 1.

### Study characteristics

**Figure 2:**
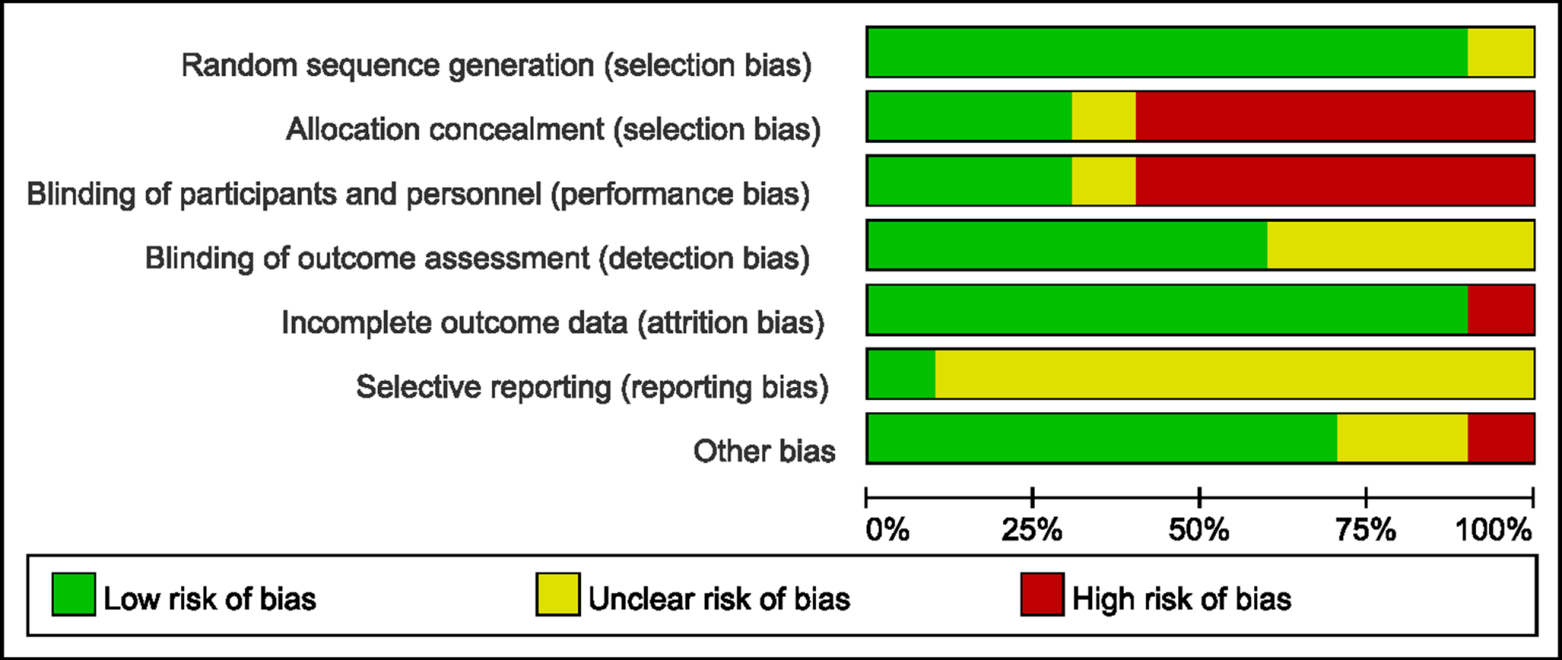
Methodological quality graph: review authors’ judgements about each risk of bias item presented as percentages across all included studies

**Figure 3:**
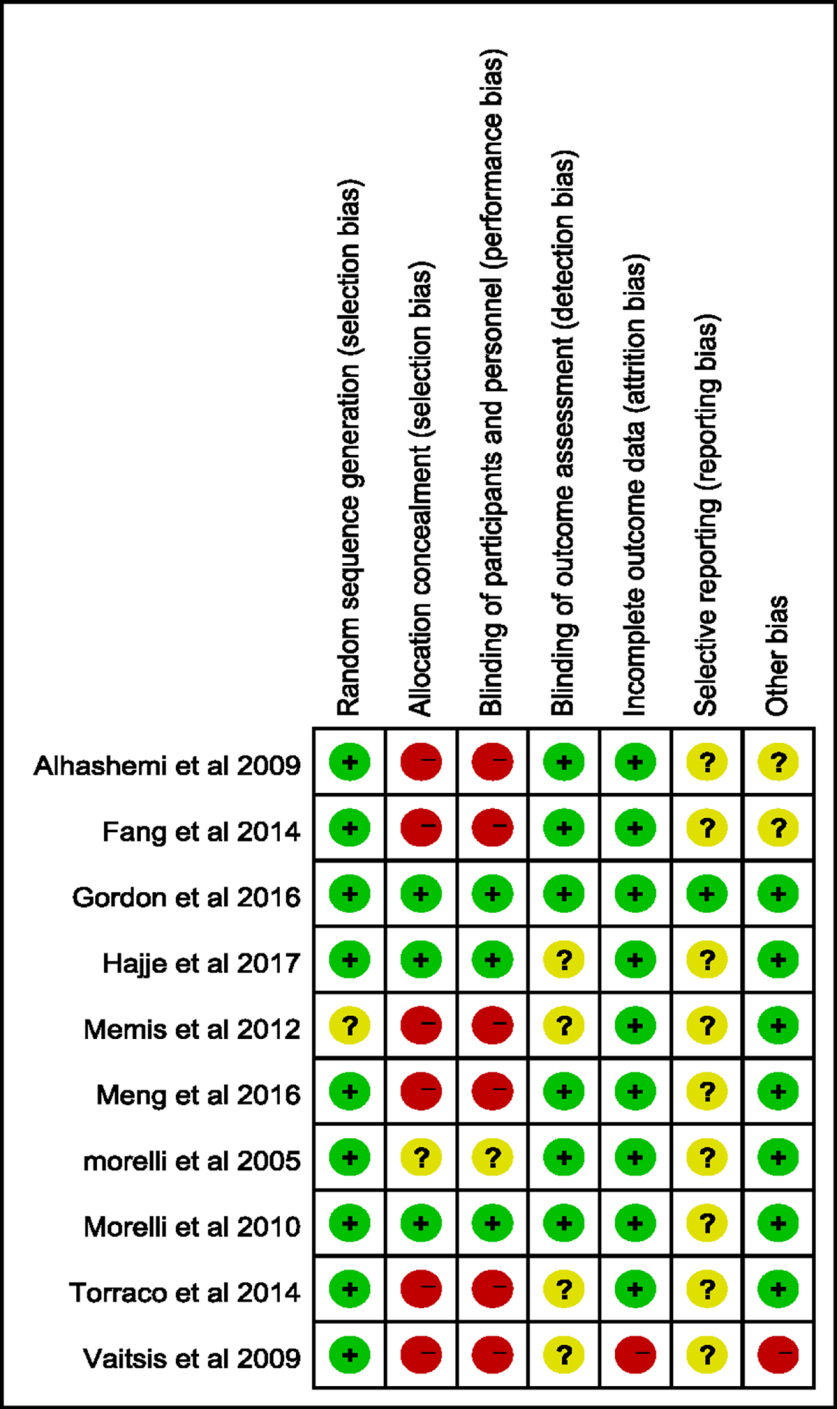
Methodological quality summary: review authors’ judgements about each risk of bias item for each included study

The main features of the ten trials included in present study were shown in Table 1. The publication year ranged from 2005 to 2017. Eight studies had dobutamine as comparator [17–19, 23, 24, 26–28], while one study evaluated levosimendan versus guideline-guided therapy [25], and another one study evaluated levosimendan versus Placebo therapy [22]. All studies administered levosimendan as a 24 hours continuous infusion without bolus. The RCT quality evaluation standard described in the Cochrane Review Handbook was used. The assessments of risk of bias for the included studies were shown in Figures 2 and 3.

**Table 1:** The main characteristics of 10 RCTs included in this meta-analysis

Study: first author	Year	Mean age of participants levosimendan/ control (years)	Setting	Levosimendan Patients	Control Patients	Control	Assessment of cardiac function	Levosimendan Infusion Dose μg/kg/min	Dobutamine Infusion Dose μg/kg/min	Lenght of levosimendan infusion
Alhashemi et al^31^	2009	NR	Severe Sepsis or Septic Shock	21	21	Dobutamine	NR	0.05–0.2	5–20	24 h
Fang et al^29^	2014	61.4/61.7	Septic Shock	18	18	Dobutamine	CI, LVSWI, LVEDI, LVESI, LVEF	0.2	5	24 h
Gordon et al^21^	2016	67/69	septic shock	258	256	Placebo	CI, SV	≤0.2	NR	24 h
Hajje et al^26^	2017	61/51	septic shock	10	10	Dobutamine	CI	0.2	5	24 h
Memis et al^19^	2012	54.93/56.27	Septic Shock	15	15	Dobutamine	NR	0.1	10	24 h
Meng et al^27^	2016	55.4/50.2	septic shock	19	19	Dobutamine	CI, LVSWI, LVEF	0.2	5	24 h
morelli et al^17^	2005	61.5/62.4	Septic Shock	15	13	Dobutamine	RAP, PAOP, SI, CI, LVSWI	0.2	5	24 h
Morelli et al^18^	2010	68/66	Septic Shock	20	20	Dobutamine	CI, RAP, PAOP, LVSWI	0.2	5	24 h
Torraco et al^28^	2014	70/68	Septic Shock	13	13	Standard Therapy	NR	0.2	NR	24 h
Vaitsis et al^30^	2009	66.1	Severe Sepsis or Septic Shock	23	19	Dobutamine	CI, EF	0.1	5–10	24 h

CI cardiac index, LVSWI left ventricular stroke work index, RAP right atrial pressure, PAOP pulmonary artery occlusion pressure, SV stroke volume, SI stroke index, EF ejection fraction, LVEDI left ventricular end-diastolic volume index, LVESI left ventricular end-systolic volume index, NR not report.

### Comparative analysis of mortality

**Figure 4:**
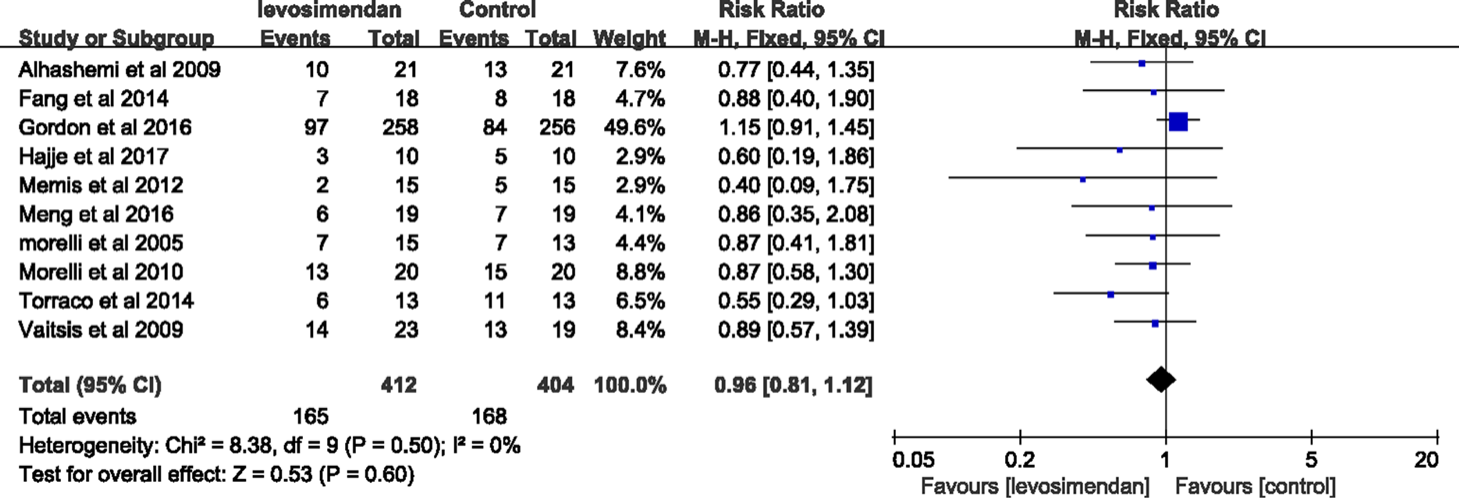
Forest plot for the risk of mortality at longest follow up available

The pooled results from the fixed-effects model combining the risk ratio (RR) for mortality were shown in Figure 4. There was no significant difference in the mortality at the longest follow-up available between the levosimendan group and the standard inotropic therapy group, 165 of 412 in the levosimendan group and 168 of 404 in the control group [RR = 0.96, 95% CI (0.81–1.12), *p* = 0.60], and the value of *I*^2^ index was 0%.

### Reliability and conclusiveness of composite outcome result

**Figure 5:**
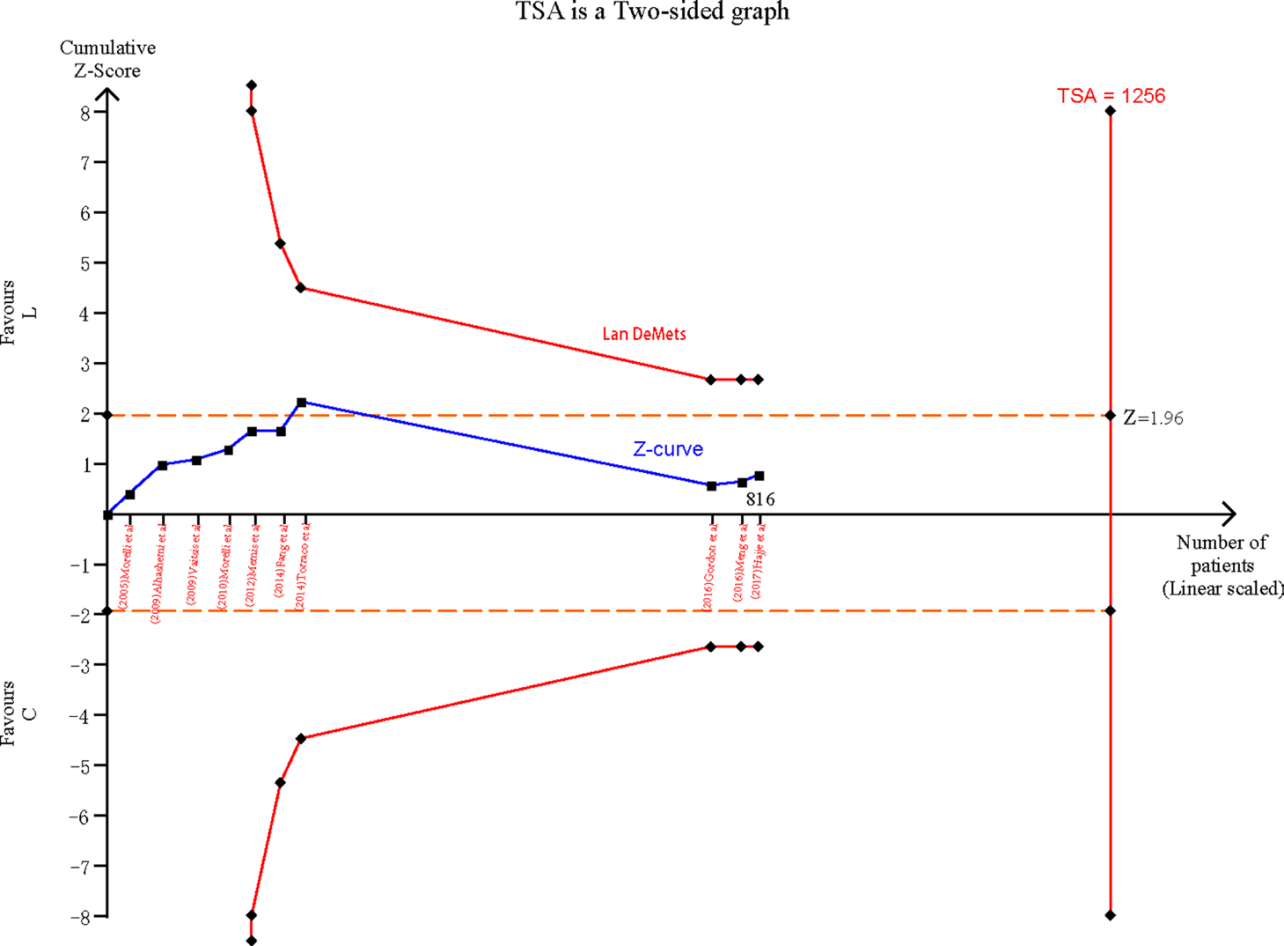
Cumulative meta-analysis assessing the effect of levosimendan on mortality in patients with septic shock

We assumed a 40% control event rate (the control event rate in our meta-analysis for the composite outcome) and a 20% relative risk reduction (the average relative risk reduction among previous trials) with 80% power and a 0.05 two sided α to determine the optimal information size. Our calculations indicated that the optimal information size needed to reliably detect a plausible treatment effect was 1256 patients. Currently, 816 patients were randomly assigned in the trials, not yet up to the size. We constructed the TSA to determine the reliability of the outcomes (Figure 5). Z value of 7 trials which published before 2015, crossed the traditional boundary, but the sequential monitoring boundary had not been crossed and the accumulated amount of information was not up to the optimal information size. These indicated that the evidence may be false positive. Combining the recent trials [22–24], cumulative Z value of all 10 trials did not cross the traditional boundary and sequential monitoring boundary, similarly the accumulated amount of information was not up to the optimal information size, indicating that the cumulative evidence was unreliable and inconclusive.

### Comparative analysis of secondary endpoints

Seven studies [17, 18, 22–24, 26, 28] reported blood lactate which was significantly reduced in the levosimendan group (RR: −0.71, [95% CI: −1.36, −0.06], *p* for effect = 0.03). There was no difference in MAP [17–19, 22–26] (RR:1.99, [95% CI:-0.66,4.64], p for effect = 0.14), CI [17, 18, 22–24, 26] (RR:0.23, [95% CI: −0.22, 0.69], p for effect = 0.32), norepinephrine dose [17, 18, 22–24, 26] (RR:0.00, [95% CI: −0.02, 0.02], p for effect = 0.88) and length of ICU stay [18, 19, 22, 24] (RR: −2.67, [95% CI: −6.18,0.85], *p* for effect = 0.14) (Table 2).

**Table 2:** Secondary endpoints after randomizations

Secondary outcomes	Number of studies	RR (95% CI)	*P* (heterogeneity)	*I*^2^ (%)	*P* (overall effect)
Cardiac index (L/min/m2)	6	0.23 [−0.22, 0.69]	< 0.00001	93	0.32
Mean arterial pressure (mmHg)	8	1.99 [−0.66, 4.64]	< 0.00001	93	0.14
Blood lactate (mmol/L)	7	−0.71 [−1.36, −0.06]	< 0.00001	94	0.03
Norepinephrine dose (μg/kg/min)	6	0.00 [−0.02, 0.02]	0.91	0	0.88
Length of ICU stay (days)	4	−2.67 [−6.18, 0.85]	0.16	42	0.14

## DISCUSSION

In the present meta-analysis, our findings demonstrated that levosimendan treatment may not reduce mortality in patients with septic shock. However, the Lan-DeMets sequential monitoring boundary which applied to TSA indicated that the cumulative evidence was unreliable and inconclusive, further studies were necessary to confirm the effect of levosimendan on mortality in patients with septic shock. Furthermore, secondary analyses showed that blood lactate was significantly reduced in the levosimendan group while there was no difference in MAP, CI, norepinephrine dose and length of ICU stay compared to control.

A recent meta-analysis [20] supported the use of levosimendan in patients with sepsis shock significantly improved the overall survival compared with that standard inotropic therapy, but the sample size was obviously too small. Previous research had shown that small studies tended to report larger beneficial effects than large trials, the conclusions of meta-analyses involving small trials may be unreliable [29]. Therefore we constructed the TSA to assess the reliability and conclusiveness of composite outcome result. *Z* value of 7 trials (i.e., included in the recent meta-analysis [20]), crossed the traditional boundary, but the sequential monitoring boundary had not been crossed. In addition, the accumulated amount of information is not up to the optimal information size, these prompted that the evidence may be false positive. Our meta-analysis which updated the data indicated there was no significant difference in the mortality between the levosimendan group and the standard inotropic therapy group. Regretfully, the cumulative evidence was unreliable and inconclusive.

From a pharmacological viewpoints, levosimendan is an inotropic agent which differs from catecholamines, such as dobutamine. By sensitizing cardiac cell to exist levels of intracellular calcium, an increase in the force of contraction during systole without affecting diastolic relaxation [12]. As levosimendan doesn’t increase myocardial oxygen demand, relaxation of the myocardium is not impaired, which may be an additional benefit over catecholamines [30]. In addition, levosimendan has an active metabolite, which has a long half-life of around 80h, a single 24-hour infusion lasts for almost a week [31], which is long enough to support the majority of patients with septic shock until hemodynamic recovery [32]. Consequently, levosimendan may be an inviting therapy in these patients. Nevertheless, the primary outcome of this study was negative, as well as the most secondary endpoints. Besides, the large randomized controlled trial [22] indicated that levosimendan was associated with a higher risk of supraventricular tachyarrhythmia compared to control and another severe side effect of levosimendan, such as severe vasoplegia that might be difficult to control, should be in attention [21].

A major strength of our study is that data were from good quality studies. Besides, we assessed mortality which was the most important clinical outcome. Moreover, we used the optimal information size to help to construct the TSA to assess the reliability and conclusiveness of composite outcome result.

Several limitations should be acknowledged as well. First, in addition to the study [22], the rest of the dataset was quite small. Second, the assessment of cardiac function in the study was inconsistent, especially the study [22] which recruited a wide range of patients with sepsis, without requiring a low cardiac output as an enrollment criterion. Third, a comparison of levosimendan with an alternative inotrope was different, eight out of the ten RCTs included in the present study used dobutamine as control inotropic agent [17–19, 23, 24, 26–28]. One study [25] evaluated levosimendan versus guideline-guided therapy, and another one study [22] evaluated levosimendan combined with guideline-guided therapy versus placebo combined with guideline-guided therapy which was different from other trials.

## CONCLUSIONS

Findings from this meta-analysis demonstrated that levosimendan treatment may not reduce mortality in patients with septic shock. The result remains inclusive and further randomized controlled trials were needed to confirm these conclusions.

## MATERIALS AND METHODS

### Literature search

Preferred Reporting Items for Systematic Review and Meta-Analyses (PRISMA) statement was followed [33]. We searched PubMed, EMBASE, Cochrane Library Database updated to March 27, 2017, without language restrictions. By following search terms: “levosimendan”, “levosimedan”, “Sepsis”, “Septicemia”, “Septic shock”, “randomized clinical trial” and so (Supplementary Table 1). Furthermore, we screened reference lists of obtained publications and reviews for additional eligible studies.

### Study selection

All relevant information in included studies was extracted by one reviewer (B. J. W), and checked for accuracy independently by another reviewer (R. J. C). They screened the titles or abstracts, or both, of the search results and assessed the remaining full-text articles for eligibility. Any uncertainty regarding eligibility was resolved by discussion. Studies were included for our meta-analysis if:(1) the study was conducted in patients with septic shock, (2) the intervention was levosimendan, (3) the outcome of interest was mortality, and(4) the study design was a RCT(i.e., not comments or review); Exclusion criteria: nonintravenous administration of levosimendan, duplicate publications and pediatric studies.

### Data extraction

Two investigators (B. J. W and R. J. C) extracted the following data: name of first author and year of publication, mean age of participants, clinical setting, sample size, control treatment, assessment of cardiac function, levosimendan dose and length (Table 1). The primary endpoint was mortality at the longest follow-up available. The secondary endpoints were cardiac index(CI), mean arterial pressure (MAP), blood lactate, norepinephrine dose and length of ICU stay. If the data were unavailable, we corresponded with the author(s) for the relevant data.

### Quality assessment

Two reviewers (B. J. W and R. J. C) independently screened the literature and assessed the quality of the literature and cross-checked. The consensus process to resolve disagreements required researchers to discuss the reasoning for their decisions, if it could not be reached, we consulted a third reviewer (Y. Y. G). Methodological quality evaluation was using the RCT quality standard of Cochrane Review, Handbook. We considered each question and classified it as “low,” ”high,” or “unclear”, such as selection bias (random sequence generation, allocation concealment), performance bias (blinding of participants and personnel), detection bias (blinding of examiner), attrition bias (loss to follow-up, incomplete outcome data), and selective outcome reporting and other bias [34].

### Statistical analysis

All outcomes were expressed as relative risk (RR), and forest plots were produced to visually assess the RR and corresponding 95% confidence interval (CI) across studies [35]. The *I*^2^ and χ^2^ would be used to express statistical heterogeneity in the meta-analysis. A useful statistic for quantifying heterogeneity is *I*^2^ = (Q − df)/Q 100% where Q was the chi-squared statistic and df was its degrees of freedom, and its ranges from 0 to 100%. Studies with an *I*^2^ statistic of 0–25% were considered to have little heterogeneity, 25–50% represented low heterogeneity, 50–75% referred to as moderate estimates, and over 75% described considerable heterogeneity [36]. We pooled the study-specific estimate using the inverse variance method and a fixed effect model. All statistical analyses were performed using Review Manager (version 5.3 for Windows; the Nordic Cochrane Centre, Copenhagen, Denmark). All statistical tests were 2-sided and α < 0.05 was considered statistically significant level [37].

The estimation of sample size was required by the repeatability principle of clinical trials aiming at the study power in meta-analysis. Therefore the TSA was used to assess the reliability and conclusiveness of the available evidence on levosimendan [38], focusing on the composite outcome of mortality in patients with septic shock. We calculated the sample size (optimal information size) requirement for our meta-analysis, and used this monitoring boundary as a way of determining whether the evidence in our meta-analysis was reliable and conclusive. TSA software we used was from the Copenhagen Trial Unit(http://www.ctu.dk/tsa/).

## SUPPLEMENTARY MATERIALS TABLE



## Abbreviations

CIs: confidence intervals
RR: relative risk
TSA: trial sequential analysis
RCT: randomized controlled trial
CI: cardiac index
MAP: mean arterial pressure
ICU: intensive care unit
