# Supplementary material for: Effects of levosimendan on mortality in patients with septic shock: systematic review with meta-analysis and trial sequential analysis
Source: Oncotarget. 2017 Aug 10;8(59):100524–32. doi: 10.18632/oncotarget.20123 (PMC5725040; doi:10.18632/oncotarget.20123)
Supplement: Supplementary file 1 [file oncotarget-08-100524-s001.pdf]

Effects of levosimendan on mortality in patients with septic shock: systematic review with meta-analysis and trial sequential analysis

SUPPLEMENTARY MATERIALS

Supplementary Table 1: Searching strategy in each database

Searching strategy
Pubmed<br>Search (((((randomized controlled trial [pt]) OR (controlled clinical trial [pt]) OR (randomized [tiab]) OR (placebo [tiab]) OR (drug therapy [sh]) OR (randomly [tiab]) OR (trial [tiab]) OR (groups [tiab])) AND (humans [mh])))) AND ((((((((((((calcium adj2 sensiti*)) OR inodilator*[Title/Abstract]) OR (((inotropic adj2 (agent* or drug* or medicat* or act*))))) OR Cardiotonic Agents[Title/Abstract]) OR simdax[Title/Abstract]) OR "or 1259") OR or1259) OR levosimedan[Title/Abstract]) OR levosimendan[Title/Abstract])) AND ((((((Sepsis) OR (Septicemia) OR (Blood stream infection) OR (Septic shock) OR (Endotoxic Shock) OR (Toxic Shock) OR (Severe sepsis))))) OR "Sepsis"[Mesh]))
Cochrane<br>#1 levosimendan or levosimedan or or1259 or "or 1259" or simdax:ti,ab,kw (Word variations have been searched)<br>#2 MeSH descriptor: [Cardiotonic Agents] this term only<br>#3 (inotropic near/2 (agent* or drug* or medicat* or act*)):ti,ab,kw (Word variations have been searched)<br>#4 inodilator*:ti,ab,kw (Word variations have been searched)<br>#5 (calcium near/2 sensiti*):ti,ab,kw (Word variations have been searched)<br>#6 #1 or #2 or #3 or #4 or #5<br>#7 MeSH descriptor: [Sepsis] explode all trees<br>#8 MeSH descriptor: [Shock, Septic] explode all trees<br>#9 (sepsis or septic* or blood?stream infection* or (shock adj3 (endotoxic or toxic))):ti,ab,kw (Word variations have been searched)<br>#10 #7 or #8 or #9<br>#11 #6 and #10
Embase<br>#17#14 AND #15 AND #16<br>#16'randomized controlled trial'/exp<br>#15'sepsis/' OR 'septicemia/' OR 'bloodstream infection/' OR 'septic shock/' OR '(sepsis or septic* or blood stream infection*' OR '(shock adj3 (endotoxic or toxic)))':ab,ti<br>#14#1 OR #5 OR #6 OR #7 OR #8 OR #9 OR #10 OR #11 OR #12 OR #13<br>#13'(calcium adj2 sensiti*)':ab,ti<br>#12'inodilator*':ab,ti<br>#11'(inotropic adj2 (agent* or drug* or medicat* or act*))':ab,ti<br>#10'cardiotonic agent':ab,ti<br>#9simdax:ab,ti<br>#8levosimendan:ab,ti<br>#7levosimedan:ab,ti<br>#6'or 1259':ab,ti<br>#5'or1259':ab,ti<br>#1'levosimendan'/exp
